# Geological controls of giant crater development on the Arctic seafloor

**DOI:** 10.1038/s41598-020-65018-9

**Published:** 2020-05-21

**Authors:** Malin Waage, Pavel Serov, Karin Andreassen, Kate A. Waghorn, Stefan Bünz

**Affiliations:** CAGE – Centre for Arctic Gas Hydrate, Environment, and Climate, Department of Geosciences, UiT the Arctic University of Norway, 9037 Tromsø, Norway

**Keywords:** Climate-change impacts, Natural hazards, Geodynamics, Geophysics

## Abstract

Active methane seepage occurs congruent with a high density of up to 1 km-wide and 35 m deep seafloor craters (>100 craters within 700 km^2^ area) within lithified sedimentary rocks in the northern Barents Sea. The crater origin has been hypothesized to be related to rapid gas hydrate dissociation and methane release around 15–12 ka BP, but the geological setting that enabled and possibly controlled the formation of craters has not yet been addressed. To investigate the geological setting beneath the craters in detail, we acquired high-resolution 3D seismic data. The data reveals that craters occur within ~250–230 Myr old fault zones. Fault intersections and fault planes typically define the crater perimeters. Mapping the seismic stratigraphy and fault displacements beneath the craters we suggest that the craters are fault-bounded collapse structures. The fault pattern controlled the craters occurrences, size and geometry. We propose that this Triassic fault system acted as a suite of methane migration conduits and was the prerequisite step for further seafloor deformations triggered by rapid gas hydrate dissociation some 15–12 ka BP. Similar processes leading to methane releases and fault bounded subsidence (crater-formation) may take place in areas where contemporary ice masses are retreating across faulted bedrocks with underlying shallow carbon reservoirs.

## Introduction

Manifestations of past and present seabed methane discharge, such as seafloor pockmarks^1^, gas hydrate pingos^2^, craters^3^, and mounds^4,5^ appear widely along Arctic continental margins. While pockmarks have received considerable attention from the fluid flow research community and petroleum industry for many decades^6–9^, km-wide seabed craters and associated mounds have garnered less scrutiny. Various studies report craters appearing on the seafloor on the Arctic continental shelf^3,5,10^, in the Scotia Sea^11^, at the Chatham Rise offshore New Zealand^12^, and on buried glacial surfaces in several areas of the Arctic^13,14^. These reports may reveal that such features are more widespread than previously thought. As opposed to the crater site at the Scotia Sea and Chatham Rise, the crater area in the Barents Sea emits methane gas from the seabed today. Andreassen, *et al*.^5^, and Long, *et al*.^15^, document spatial relationships between craters and gas expulsions, and hypothesize that the craters are collapse features caused by blow-outs of over-pressured methane accumulations. A causal relationship between methane dynamics and crater development, however, remains unclear.

Methane seepage from the seabed is the largest source of methane to the ocean^16^. Nevertheless, due to methane dissolution and subsequent aerobic oxidation in the water column e.g.^17–20^, methane from deep water seep sources contributes less to the atmospheric carbon budget than previously thought. Because the lifetime of a methane bubble in the ocean depends on how much methane is already dissolved in the water and for how long the bubble is exposed, shallow-water seeps and larger plumes of methane gas have higher potential to reach the atmosphere. Gas blowouts from the relatively shallow-water Barents Sea region (~330–350 m) or in form of more dramatic expulsions may expulse methane gas at quantities and rates sufficient to pass through the water column avoiding its complete dissolution and microbial degradation^21^. Therefore, understanding the formation of the seabed craters studied here may shed more light on processes of seabed gas emissions, and, therefore, their potential to reach the atmosphere.

During the last glacial cycle (~35 ka BP until today) the Barents Sea experienced large variations in temperature, pressure and isostacy due to growth and retreat of a large grounded ice sheet complex^22^. At the crater field in Bjørnøyrenna (Bjørnøya Trough), the ice thickness reached ~1.5 km and generated an overburden pressure of ~13 mPa, which had several important implications. First, growth and subsequent rapid retreat of large ice mass could reactivate preexisting faults^23^. Second, due to high pressure conditions during the glaciation, methane would exist the solid and highly concentrated form called gas hydrate^5^. Ice sheet retreat would cause depressurization of the subglacial strata leading to gas hydrate dissociation, free gas release and potential seepage into the water column. Third, removing the weight of the ice sheet caused isostatic adjustments of formerly depressed lithosphere, leading to gradually shallower water depth.

Investigating the evolution of pressure and temperature conditions in Bjørnøyrenna during the last 30 ka, Andreassen, *et al*.^5^ found that upon ice sheet retreat some 16 ka BP and until around 10 ka BP, the thickness of the zone, where gas hydrates structure I and II could occur, shrunk from ~500 m to 0 m (structure 1 hydrate), or from ~700 m to 100 m (structure 2 hydrate). Gas release due to gas hydrate dissociation is hypothesised forming over-pressured accumulations sealed by the remaining hydrate layer. Progressive growth of the gas reservoirs and thinning of the hydrate seal is hypothesized to eventually have led to gas blow out events forming the craters. However, to date there is no direct empirical evidence for gas blow outs nor bedrock collapse. Previously published 2D seismic data could not reveal subseafloor architecture beneath the craters in detail due to data limitations.

**Figure 1:**
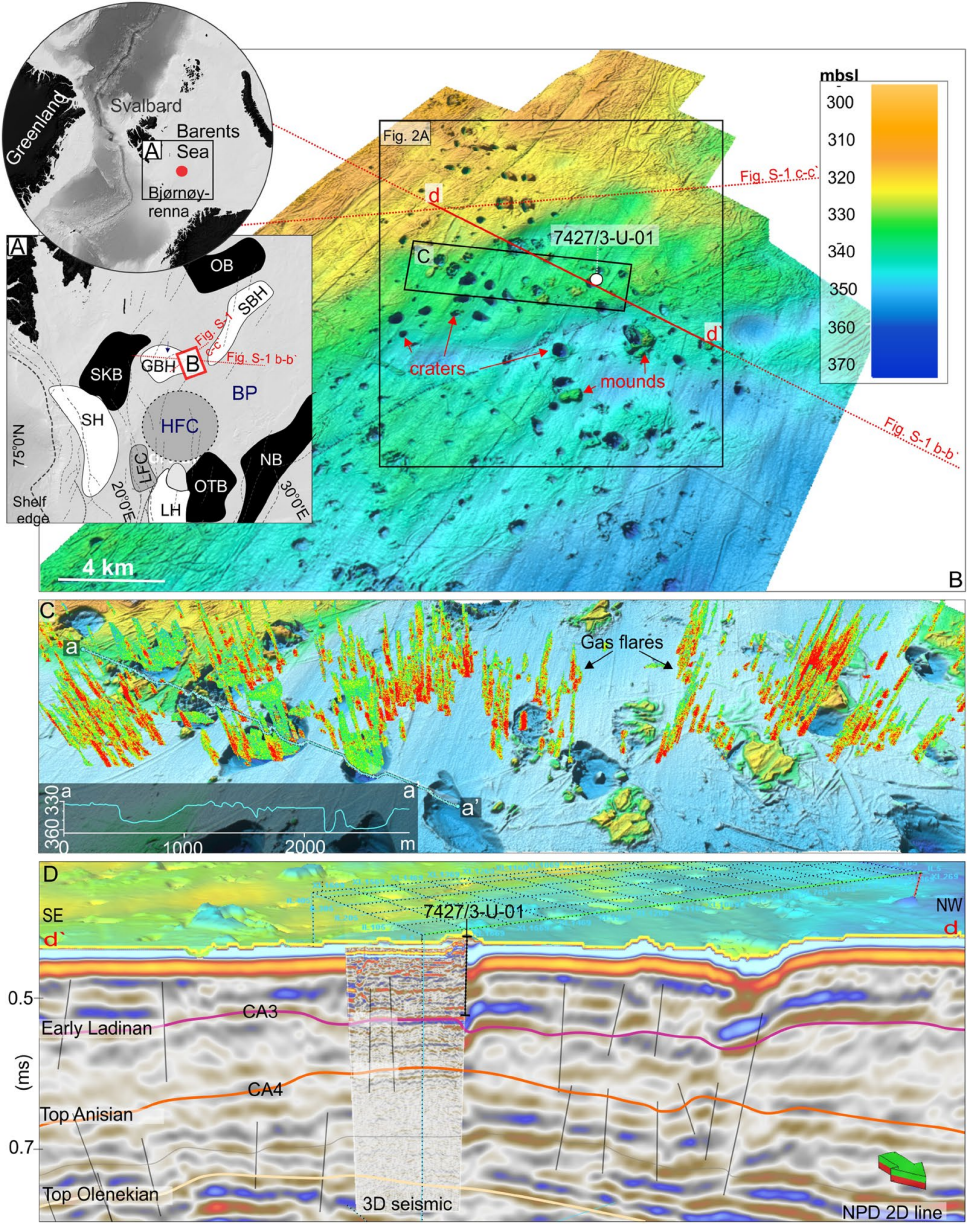
Overview of the study area. (**A**) Location of the study area (**B**) on a regional bathymetric map. (**B**) High resolution bathymetry of the Crater and Mound area. Red lines mark the location of seismic line shown in (**D**), and regional seismic lines with published interpretation shown in the supplementary. Black areas outlines regional basins, and white areas structural highs: OB - Olga Basin, SBH - Sentralbanken High, GBH - Gardarbanken High, SKB - Sørkapp Basin, HB - Hammerfest Basin, LH - Loppa High, OTB - Ottar Basin, NB - Nordkapp Basin, SH - Stappen High, BP – Bjarmeland Plattform, LFC - Leirdjupet Fault Complex 20. HFC – Hoop Fault complex 21. B - High-resolution seafloor topography of study area showing craters and mounds. The squared area delineates the bathymetric data used in this work for statistical investigations of crater morphology. (**C**) Indicates locations of the 3D seismic data set, and red line indicate location of 2D seismic line presented in (**D**). Seafloor bathymetry is acquired with a multibeam echosounder. C- Zoom in of 3D seismic area showing high-resolution bathymetry and echosounder data indicating areas of gas seepage. D - d-d‘ show an example of the seismic stratigraphy and demonstrate the detailed resolution of the P-Cable seismic data compared to conventional 2D lines across the area.

Here we use new P-Cable 3D seismic data aiming to provide crucial information on the subseafloor 3D architecture of the craters and mounds in the Barents Sea (Fig. 1), the nature of deformation within the sedimentary bedrocks and the relationship between faults and topographic features, and contemporary methane release. The 3D seismic data images the top 150 m in great detail with a horizontal and vertical resolution of ~6 and 3–5 m, respectively (Fig. 1d-d‘ shows a seismic example and C outlines area of 3D seismic). Furthermore, seafloor bathymetry data of 5 m resolution is provided by a 30-kHz Multibeam Echosounder, and water-column gas flare data by a 38-kHz Singlebeam Echosounder (see methods chapter). Combined, these datasets add important additional information to the study by Andreassen, *et al*.^5^ making progress towards understanding the nature of, and role of methane dynamics in seabed crater development in polar regions.

## Geologic and physiographic setting

The northern Barents Sea is an intracratonic sedimentary basin that has gone through several shifts in stress regime due to variations in rifting, folding, uplift, subsidence and erosion leading to the present configuration of basins, highs and platforms^24,25^. The study site is located on the SE flank of the Gardarbanken High (Fig. 1A) initiated due to rifting in Carboniferous. The faults confining the high (Figs. 1d-d, S-1A) were reactivated in late Permian-early Triassic in response to flexural upwrap^25,26^. Onlap and truncation structures of Permian carbonates and Triassic siliciclastic strata on top of the high suggest periods of subaerial exposure^5,27^ (Fig. S-1A). The greater Barents Sea was predominantly influenced by episodes of rifting during the late Paleozoic and Mesozoic. The Mesozoic Hoop Fault Complex (HFC), located 100 km south of our study area (Fig. 1A), is the nearest area where faults in Triassic rocks are studied in detail (mapped using 10,000 km^2^ 3D seismic data). There, Collanega, *et al*.^28^ revealed densely spaced (1–2 km) extensional fault pairs, which are oriented NNE-SSW and NW-SE. The fault pattern defines a system of small horsts and grabens^28^. Based on seismic observations such as thickness variations indicating ongoing tectonism during deposition led to their conclusion that the faults are of early Triassic age^28^.

Ultra-high resolution (Sparker) data, sediment sampling and ROV video surveys point towards <2 m thick veneer of unlithified glacial and postglacial deposits in our study area^5^. The subcropping Triassic (Early Ladinian) rocks are evident from a shallow stratigraphic borehole (7427/3-U-01) located within our 3D seismic area and covering the upper 88.8 m of the strata (Figs. 1, S-1A). The early Ladinian mudstones contain several 5–20 m thick coarsening upward units with hummocky cross bedding interpreted as pro-delta deposits of Snadd formation^29^ (Fig. 1E). Beyond that, the industry 2D seismic reveal that the uppermost 600 ms (~600 m, assuming an interval velocity of 2000 m/s) of the study area show the bottom sets, foresets, offlap break and proximal top sets of clinoforms of Induian to early Ladinian age^27^.

Methane gas seeping from the seafloor is likely sourced by a shallow petroleum system within the mid-Triassic clinoforms^5^ since these organic rich bottom-sets are one of the most important source rock throughout the Barents Sea^29^, and moreover, the top sets are evident to contain more porous, coarser grained reservoir quality sandstones^30,31^ (Figs. 1d‘-d, S-1A-B).

## Results

### Morphology and seismic attributes of craters and mounds

**Figure 2:**
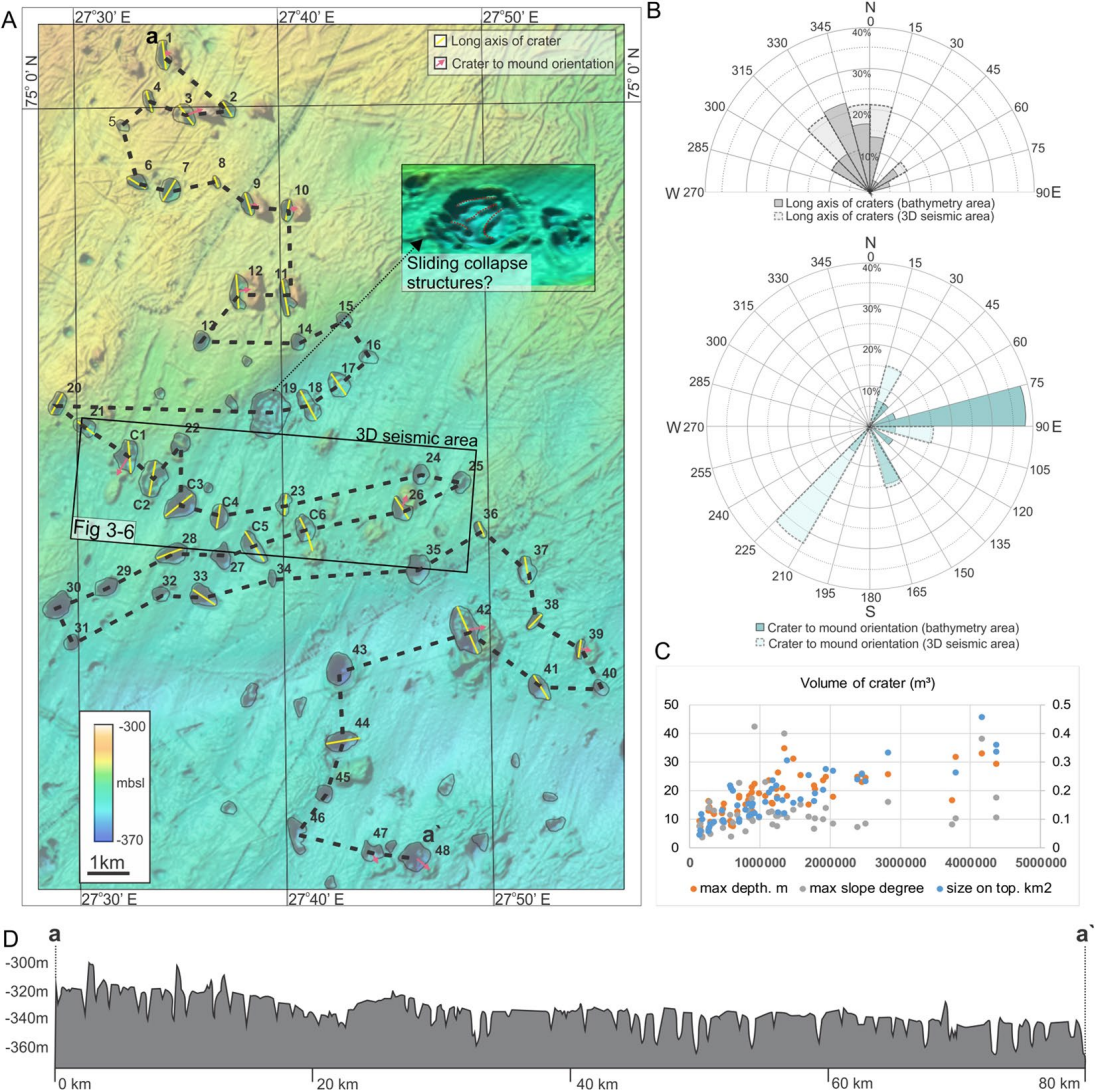
The study area with 54 large craters and 15 mounds. (**A**) bathymetric map. The yellow lines represent the long axis of craters and pink arrow the crater to mound orientation. The craters that show an asymmetry of >1.6 are regarded as elongated, and included in the orientation measures. These orientations are presented in a rose diagram in inset (**B**). The data show a NNW trend of crater orientations, and that mounds tend to be located on the eastern side of the crater-mound couples. (**C**)-panel showing statistical data where crater volume (m^3^) is plotted against surface area (m^2^), maximum depth (m) and maximum slope (degrees). (**D**) profile through all craters.

Within the study area of 710 km^2^ we observe ~100 craters with diameters ranging from 300 m to 1000 m and depths up to 35 m. Distributed around and within craters, rounded and elongated to irregular shaped mounds and pinnacles are up to 2000 m wide and 20 m tall. We focus on the central part of this area, comprising 54 pronounced craters and 15 mounds and representing the highest density of these morphological features (Figs. 1B, 2). Apart from craters, mounds, and shallow (1–3 m deep) ploughmarks, the seafloor within the study area is relatively smooth, and gradually deepens from north (325 m water depth) to south (350 m water depth). Iceberg ploughmarks are concentrated in the northern part likely due to shallower water depths and a thin drape of till deposits prone to iceberg scouring^32^.

**Figure 3:**
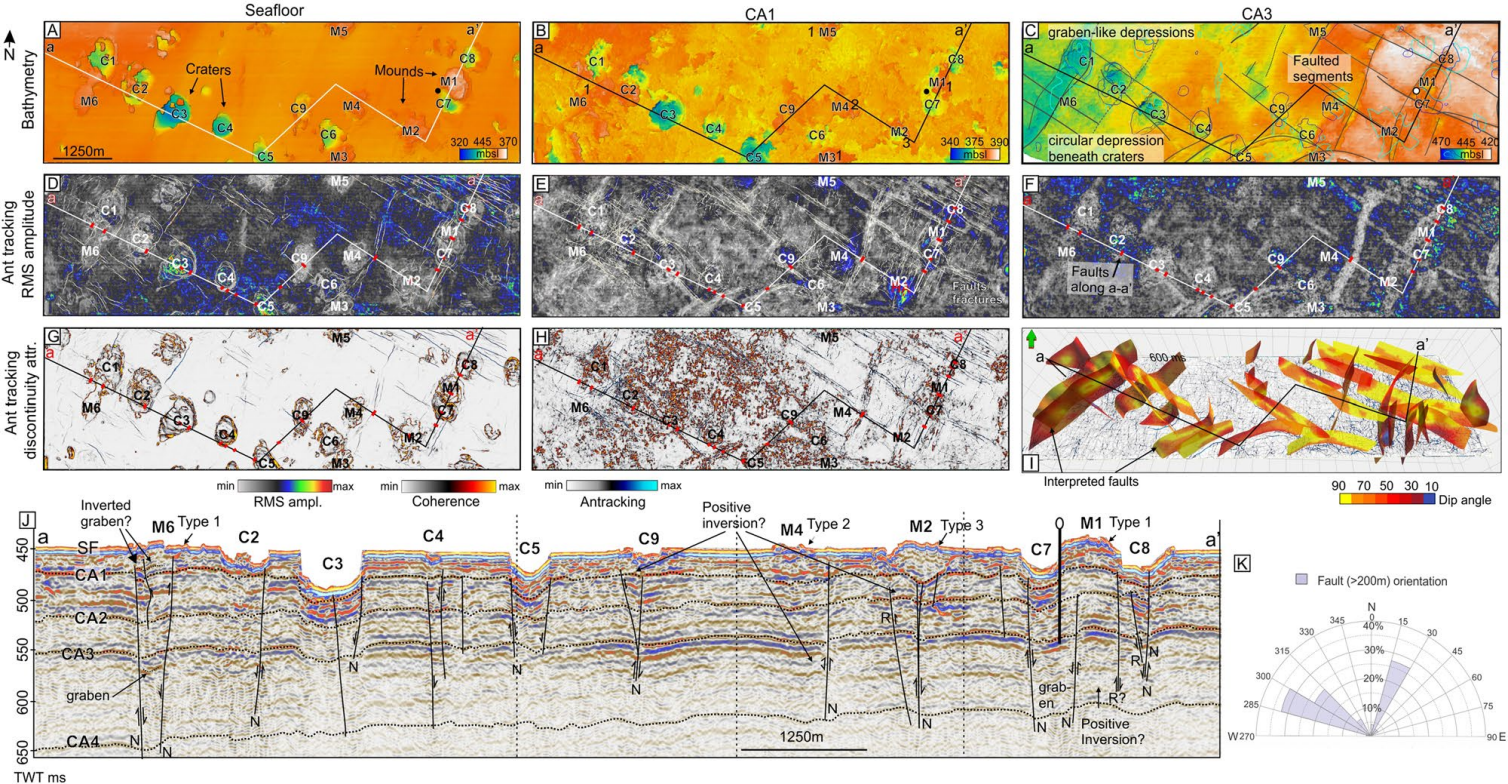
The subsurface geology beneath the seafloor is presented using depth converted surfaces (**A**–**C**) -, RMS amplitude (**D**–**F**) -, coherence and ant tracking (continuity) (**G**–**H**) of the seafloor and two deeper horizons (CA1 and CA3) I. 3D view of the manually interpreted fault planes. (**J**) a seismic line (time-depth) crossing most of the prominent craters and mounds. The seismic line shows location of horizons CA1, CA2, CA3, CA4, and examples of mound types 1, 2 and 3. C3 and C5 appear to persist down to the level of CA3. K. Rose diagram showing the fault orientations within the 3D area. Two fault orientations dominate, that is to the NW and NNE.

The craters have three types of morphologies: rounded, elongated and complex with multiple pinnacles and ridges inside. Some internal ridges curve parallel to crater walls with a gap between, showing resemblance with sliding collapse structures (Fig. 2A). Most craters have at least one or two very steep wall sides (<10–35 degrees), and a flattened base making them morphologically different from pockmarks which normally have walls that gradually flatten (~5–0 degrees) towards the centre point (Figs. 2A, 3A)^33^. The upper edges of the craters are sharp and do not exhibit rim structures or other features. The long axes of the craters show a predominantly NNW orientation (Fig. 2A,B). 20% of the craters (11 out of 48) are associated with adjacent mounds. The mounds that are part of such mound-crater pairs often have dimensions and orientation similar to the nearby crater. In the north and south of the area studied, mounds tend to occur on the eastern side of the craters while in the middle of the study area they occur on either the north or south (Fig. 2A,B). Furthermore, the craters and mounds appear slightly more elevated and narrower in the north compared to the southern area, where the craters are less elevated but wider (Fig. 3A).

A strong seafloor reflection is observed throughout the 3D area caused by the strong contrasting seismic velocity between the water column (~1470 m/s) and the hard Triassic bedrock ~1800–2200m/s. It appears that any loose sediments on top of the lithified Triassic rocks are either below the vertical resolution (~3–4 m) of the seismic seafloor throughout the 3D area, or not present.

The high-resolution 3D seismic data shows exceptionally high and continuous reflection seismic amplitudes from the bottom of the large craters, and low and discontinuous amplitudes along the mounds and inside some small craters, demonstrating a complex morphology (Fig. 3). These variations and discontinuities likely relate to differences in physical properties e.g. lithification, structure, rock strength, fluid content. Mounds and areas with a lower amplitude and lack of seismic continuity probably consist of a more fractured and weaker material, leading to lower acoustic impedance contrasts. Crater bases showing higher seismic amplitude and better continuity might on the other hand represent harder, less reworked surfaces.

### Fault characteristics and damage zones

Previously identified subsurface reflections within the Triassic interval^27^ i.e. Top Anisian (CA4) and Early Ladinan (CA3) maximum flooding surfaces, are easily identified in the 3D seismic cube (Fig. S-1B). Within the top ~150 m vertical interval, we interpret four seismic units separated by continuous to semi-continuous surfaces: CA1, CA2, CA3 and CA4, from top-down (Figs. 1D, 3J).

**Figure 4:**
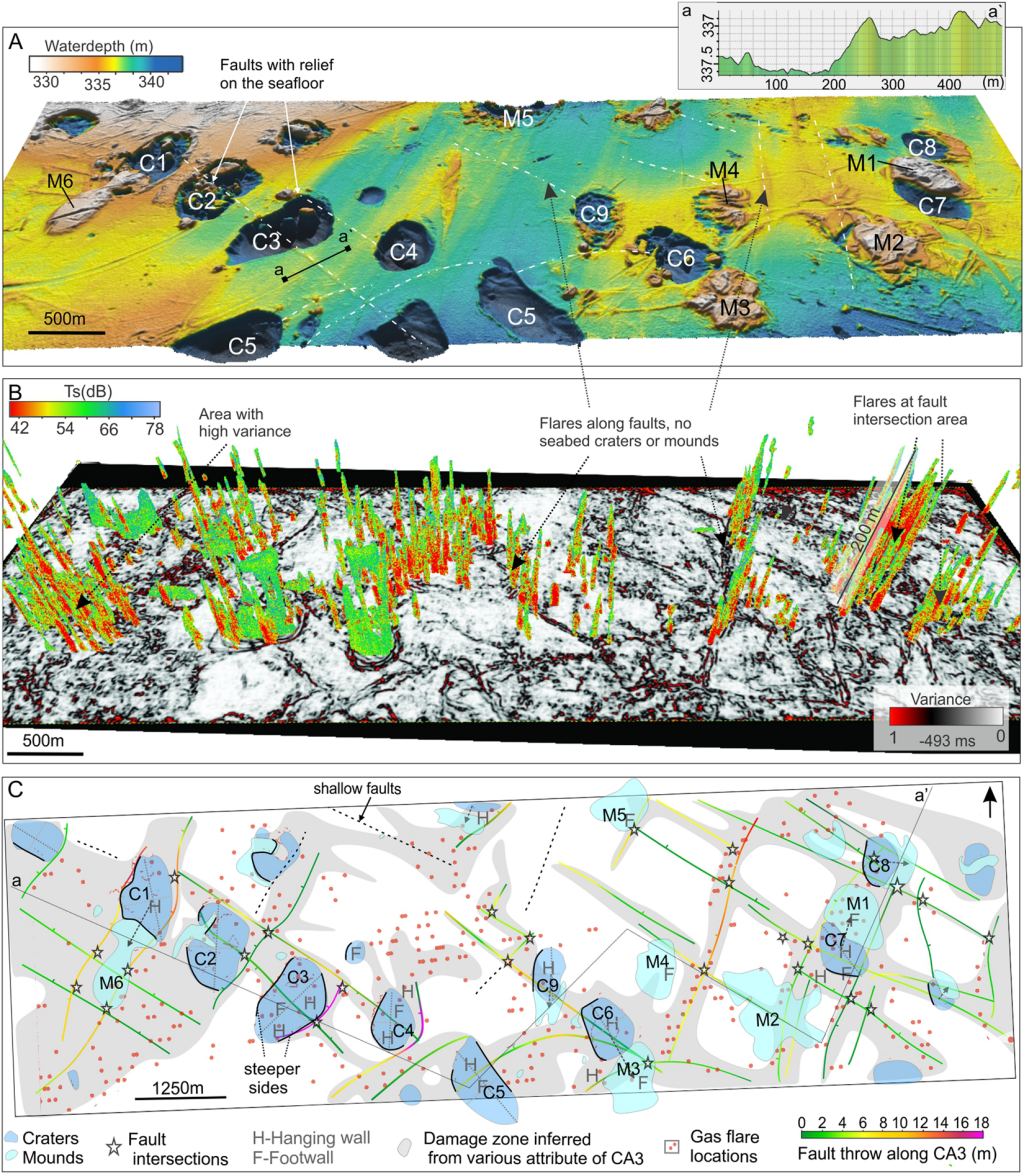
(**A**) High-resolution bathymetry of the seabed (depth below sea level) revealing small fault-related relief within the 3D study area. (**B**) Variance map of a time-slice at 493 ms TWT that is ~ 40–50 m beneath the average seafloor, indicating fault lineaments in the sub-seabed. Flares typically occur from areas of high variance, such as above craters and mounds and along the fault zones. (**C**) map view of CA3 showing location and orientation of interpreted faults, their intersection points (purple stars), and damage zones mapped from the various attribute maps around CA3, and surface craters and mounds and their orientations as well as location of gas flares. Coloured lines indicate magnitude of fault displacement.

Throughout these surfaces, the 3D seismic data reveal a dense (<2 km apart) network of faults and fractures (discontinuities without visible fault displacements) between the seafloor and the maximum imaged depth. The faults demonstrate two strike directions perpendicular to each other: 15–30° (NNE) and 285–315° (NW-WNW) (Fig. 3), causing the sedimentary rocks to split into a mosaic of blocks. Most faults appear purely extensional, however some faults show extensional characteristics in the deeper parts and compressional characteristics in the shallower parts, i.e., beneath M6, M4 and mound beside C9 (Fig. 3J), which may indicate that these are positive inversion structures (extensional faults reactivated as reverse faults). A few faults appear purely reverse, i.e., fault NW of mound M2 and fault between M1 and C8 (Fig. 3J). In the southern part of the 3D seismic area, faults with non-linear fault planes dip south. Fault dips are generally steep, varying between 50–90 degrees assuming a subsurface velocity of 2000–2200 m/s (Fig. 3I). Faults striking NW-SE generally have smaller fault displacement, up to ~8 m, than faults striking NNE-SSW which have a displacement of up to ~16 m (Fig. 4C,K). Fault displacements along horizons are largest at the deepest horizon CA4 and become gradually less towards the shallowest horizon (CA1), which does not show seismically resolvable displacements (apart from beneath the craters and across mounds) (Fig. 3B compared to C, J, and S-2). Yet, multibeam bathymetry data with higher vertical resolution than 3D seismic reveal that several faults are displaced <40 cm on the seafloor (Fig. 4).

Zones of low seismic amplitude (Fig. 3D–F) and low continuity (Fig. 3G–H) correlate with fault zones and prominent fracture networks (Fig. 3I). Moreover, the ant tracking maps (attribute that detects discontinuities) indicate zones of distributed faulting around the main fault planes. These fault zones vary in extent, but are typically wider along the larger displacement faults striking NNE-SSW (up to ~250–300 m as shown on Fig. 3C (displacement along CA3), Fig. 3F–H (continuity) and Fig. 4), and at the fault intersection areas where NNE-SSW and NW-SE faults meet (up to 3–4 km, i.e. SE damage zone, Fig. 4). We interpret these areas of low amplitude and increased fracturing around fault planes and fault intersection areas as damage zones. The damage zone of a fault represents the brecciated zone^34^ naturally exhibiting lower continuity of seismic reflections and a reduction in seismic amplitude. Because of the strong spatial connection between the zones of breccia and faults, it is likely that the initial damage zones are syngeneic to faults and are of the same age. However, fault zones of 250–300 m are 25–30 times wider than what empirical studies of fault displacements versus fault zone width suggest for faults displaced 10–16 m^35^. This points towards modification and widening of damage zones after initial formation.

### Crater, mound- and subsurface strata characteristics

**Figure 5:**
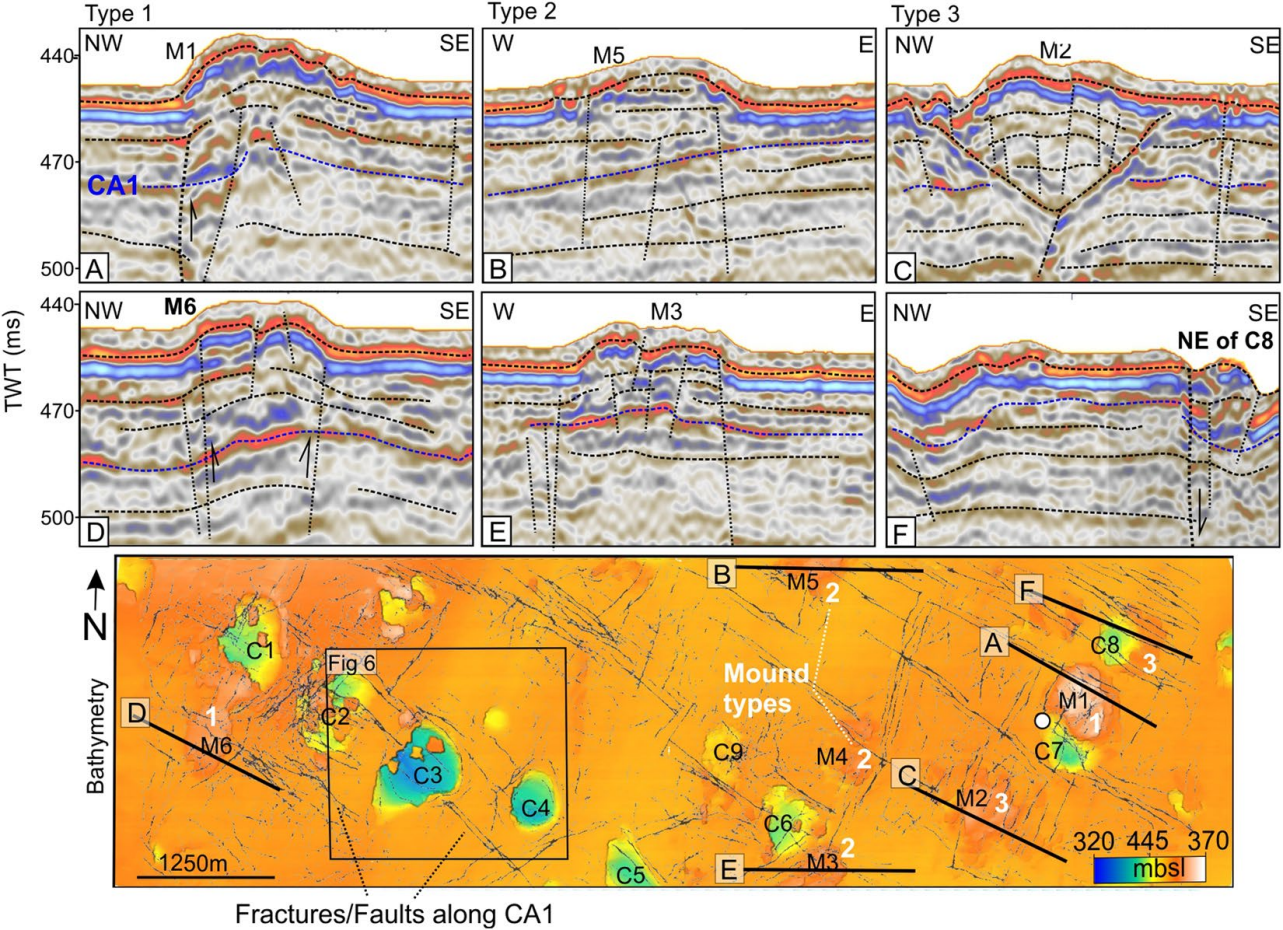
Seismic and seafloor expressions of types of mounds across the 3D seismic area. Ant track map of CA1 is draped on top of the topography map of the seafloor to indicate the shallow fracture-network. Type 1 – up-bend of sedimentary strata below mound, Type 2 – Mound appear to rest on flat surface, Type 3 – depression beneath mound.

Our 3D seismic data shows that craters are often enclosed between intersections of the NNE-SSW and NW-SE faults or confined to faulted blocks demonstrating large (<16 m) displacements and wide damage zones (Figs. 3–5). All investigated craters lie within interpreted damage zones surrounding the faults (Fig. 4). In some instances, the long-axis of craters strike at 45 degrees to both dominating fault directions (Fig. 2B versus 3 K, 4 C) and can suggest that craters develop at an oblique angle from or at the middle of intersecting faults.

The uppermost surface CA1 is relatively planar and parallel to the seafloor however the horizon and surrounding strata bend downwards below craters, or upwards, forming a positive topographic feature beneath some of the mounds (e.g. at mound M1 and M6, Figs. 3 and 5). The latter typically occurs where the mounds reveal no distinct basal seismic reflections. Furthermore, mounds M1 and M6 appear like “pop-up” blocks delimited by faults (Fig. 5A,D). We call mounds that show this characterization type 1 mounds. Other mounds such as M4 appear to rest on the seafloor reflection, and therefore show less subsurface deformation (Fig. 5B,E). We characterize such mounds as type 2. A last type 3 are mounds such as M2 that overlays a depression (Fig. 5C,F). The seismic reflections of the deeper surfaces CA2, CA3 and CA4 commonly bend down (~5–15 m on depth converted surfaces) beneath the seafloor craters and slightly upwards beneath mounds type 1 (Fig. 3J). The layered strata between the seafloor and deepest horizon (CA4) are somewhat more chaotic and fragmented than outside crater and mound locations (Fig. 3).

**Figure 6:**
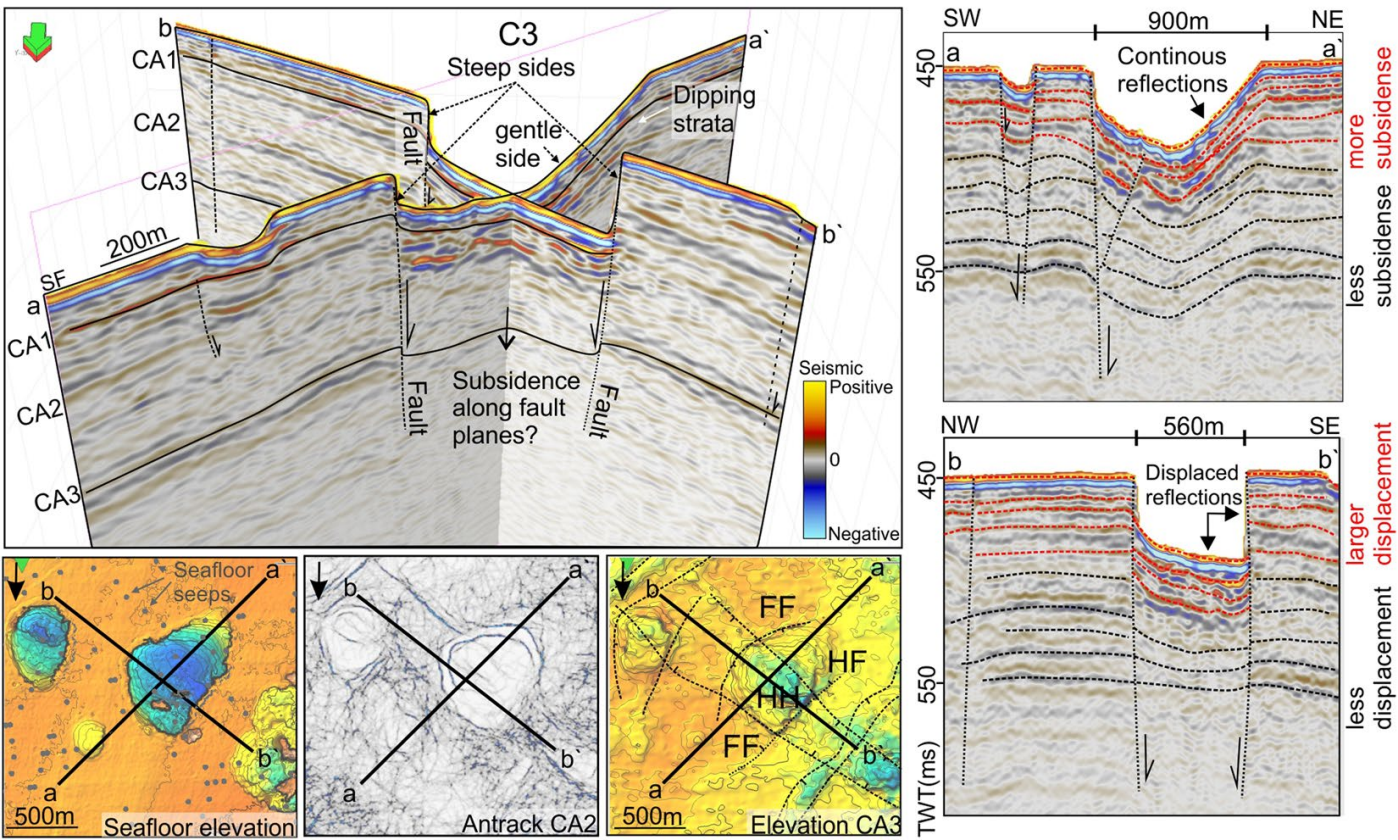
Figure illustrating fault and non-fault bounded crater-sides. In this example, three sides of the crater are steep, fault-bounded sides suggesting subsidence along fault planes, whereas the forth side (NE side) is bedrocks subsiding due to the downward movement along the other crater sides.

Fault planes coincide with one or several walls of the craters and several flanks of the mounds (Figs. 3, 4, 6). The crater walls that coincide with the fault planes typically form the steepest and the most planar side(s) of the crater. Composite seismic lines show that the crater bases and underlying shallow strata are abruptly displaced downwards (<35 m) along the fault planes forming the steepest crater walls (Fig. 6). At the gentler walls the strata gradually bend upwards towards the seafloor (Fig. 6). Therefore, craters appear as down-faulted half-grabens or grabens; depending on how many sides are fault-defined.

### Gas hydrate potential and current methane seepage

Modeling the base of the GHSZ, assuming 100% methane gas, shows that methane gas hydrate can only be stable beneath the three deepest craters^5^ given the present seafloor configuration. However heavier hydrocarbon gases were measured at the sites in the area^5^, implying there is potential for a deeper stable gas hydrate zone. The seismic data show no evidence of an extensive reflection marking the change between stable gas hydrate and free gas, also called a Bottom Simulating Reflection (BSR). However, beneath the three deepest craters, there are strong amplitude anomalies which may be related to the fluid accumulations or potentially gas hydrates.

Current seafloor seeps across the 3D seismic area are sourced from craters, mounds and from NNE-SSW and NW-SE fault zones outside of the craters or mounds (Fig. 4A,B). There are some variations in flare height (50–200 m above seafloor) and intensity across the study area. High (~200 m) intense gas flares especially rise from the wide fault zone striking NNE-SSW between mound M4 and M2, and from fault intersections or faulted blocks (Fig. 4B). Only the latter coincide with areas of seafloor craters and mounds. Distributed but less intense flaring occurs from the deeper craters where hydrate structure 1 may be stable. Apart from these observations, the strength of individual seeps does not correlate with structural or morphological elements.

## Discussion

The geophysical data used in this study show new details of giant craters and mounds that developed some 15-12 ka BP in central Barents Sea associated with the deglaciation of the area, and their subsurface characteristics, relationship to shallow stratigraphy and in particular fault-and fracture network. The study enables a better understanding of the geological controls and development of the craters and mounds and current methane seepages.

A pronounced NNW-SSE trend of crater orientation (long diameter of crater) and mounds typically appearing on the NE side of the crater indicate a uniform controlling factor on crater and mound morphology and their relative distribution. The seismic data reveal an extensive fault system, which aligns with craters, mounds and current seeps, indicating that their distribution as well as shape is controlled by NNE-SSW and NW-SE striking faults (Fig. 4C).

Collanega, *et al*.^28^ find that fault displacement reduction towards the fault tip indicated syn-depositional deformation. Here we also interpret upwards lessening of fault throw to be a consequence of syn-depositional deformation as opposed to the faults being blind faults (Fig. 4J). Moreover, the inversion structures suggest episodes of fault reactivation. Collanega, *et al*.^28^ find that the Hoop Fault Complex is a conjugate fault system caused by rifting motions in the Triassic^28^. The fault characteristics of the upper Triassic unit in our study area, fault orientation (NNE-SSW and NW-SE), density of lineaments (1-2 km), and fault displacement trends (decreasing block displacement towards younger rocks) are very similar to the same Triassic unit in the Hoop Fault Complex. From this we suggest that the craters and mounds are structurally controlled by older Triassic conjugate fault system with a similar tectonic regime as the Hoop Fault complex some 100 km further southwest.

There are a number of interesting observations regarding the fault system and crater- mound characteristics. First, fault planes strike along the corners and walls of the craters, and the craters and mounds are consistently located on top of semi-isolated blocks defined by intersecting faults. Second, sedimentary layers are relatively continuous across craters and mounds, and all craters are located on top of depressions in the subsurface, i.e. along CA3 the depressions are 5–15 m deep. These depressions are typically located in fault quadrants where both sides are interpreted as the hanging-wall (down-faulted) side. While steep sides of craters are sharp and commonly lie along fault-planes, gentler sides are often transitional with a rather planar-tilt from the crater bottom to its edge indicating failure along fault-planes and subsequent bending along other gentler side. Third, 6 of 10 mounds (type 1 mounds) are fault-bounded and appear as uplifted blocks routing at least 100–150 m deep. Lastly, the craters in our study area are not oriented along the fault axis, but are oblique to the two main fault orientations.

These observations cause craters to resemble half-grabens or grabens, and most of the mounds to resemble horsts. The crater orientation may inherit an orientation and width of the fault blocks, which will be widest along the diagonal oblique to fault pairs. Seafloor subsidence forming the craters can also explain the steep pinnacles that are left in the middle of some of the craters (representing “survived” structures), and that they are more varied in surface area than depth, contradictory to pockmarks that are often demonstrate a specific depth-volume ratio^36^.

However, there are some problems with interpreting these seafloor structures as exclusively being structural subsidence. First, the seafloor does not appear like a faulted surface because only minor fault-displacements (<40 cm) are visible. Second, the intensity of iceberg ploughmarks on top of the mounds and lower seismic amplitude across the crater and mounds features compared to surrounding seafloor suggest that they are of a softer material than the surrounding seafloor and crater-bottoms. Third and most important, the height of the mounds (<~15 m) and the depth of the craters (~10–35 m) cannot be solely explained by structural uplift and subsidence since the craters are deeper than subsidence along deeper surfaces (i.e. along CA4) and mounds are taller than uplift along the deeper surfaces.

It is however known that gas hydrate growth and dissociation are accompanied by changes in bulk volumes in sediments, loss of sediment strength and subsidence of ground surfaces^37^. Experiments of hydrate growth and dissociation in sediments have shown that the sediment volume will remain expanded compared to initial sediment volume after dissociation leaving a permanent widening of pores, fractures and fissures^38^. The conceptual model of mound growth and crater formation presented in Andreassen, *et al*.^5^ focussing on the same craters and mounds as this study, explains that during more widespread and thicker gas hydrate stability conditions during the last glaciation, growth of gas hydrates in pore spaces and fractures, exerted pressure on the sedimentary lattice. The pressure increase promoted volume expansion and build-up of mounds, as well as inhibited subsurface and seafloor fluid seepage. Andreassen, *et al*.^5^ further suggest that during the deglaciation, the thinning of the GHSZ led to methane release and sequestration in high-pressure gas accumulations underneath the hydrate cap and, eventually, methane blowouts which led to collapse of the mounds and crater formation. We build upon this model and suggest that the existing fault pattern and fracture characteristics played an important role in focussing fluid flow, increasing the accommodation space for gas hydrate growth and later methane release areas. To our knowledge, the positive topographic structures in our study are the first mounds observed within lithified bedrocks that may have formed due to fluid flow processes. As opposed to unlithified sediments, brittle deformation in layered lithified bedrock associated with strata expansions related to fluid flow processes such as gas hydrate growth will likely occur in “blockier” manner. Therefore, we suggest that fault-bounded focused seepage and gas hydrate growth in the lithified layered sedimentary bedrock have developed the mounds and made them appear like horsts or pop-up structures, and we interpret them as fault-delineated zones of uplift and expansion due to gas hydrate growth and dissociation, which, commonly are accompanied by changes in bulk volume^39^. In areas of more intense gas hydrate growth and subsequent methane release, the sedimentary lattice may collapse causing subsidence after depressurization and hydrate dissociation^40^. Hydrate dissociation is also accompanied by the release of water, a process that is known to soften sediments^40^. Gas, water and mobilized sediments might have found their way up to the seafloor through fractures and faults causing an overall volume loss at depths and a subsiding seafloor along pre-existing weakness zones, in areas where the potential for gas accumulation and hydrate growth is the largest. Most craters appear to orient in the hanging-wall-hanging-wall quadrant of a fault pair, where original fault displacement potential is the largest and fault related-damage-/brecciated zone the widest. This is expected, since fracture zones would enhance fluid flow and increase accommodation space for hydrates.

Presently gas seepage predominantly occurs from the fault zones, but also from the wider damage zone area. The abnormally large width of some of the damage-zones^35^, as well as fault displacements on the seafloor indicate the influence of fault reactivation and fluid activity. It is likely that uplift, erosion, removal of ice loading and isostatic rebound caused reactivation and inversion of faults, propagation of new fractures, as well as fluid- and gas hydrate activity. Structural fabrics and weaknesses in host rocks exacerbated these processes, and continued upwelling of gas probably initiated additional shallow fractures close to the surface, branching from the primary fault, resulting in widening of the damage zones and related seafloor structures.

We suggest that subsurface characteristics of type 3 mounds (mounds that appear to overlie a depression) are explained as low angled fractures that formed due to shallow gas escape. The fractures are likely filled with hydrate or gas causing visible reflections. An indication that the mound type 3 is not composed of ejected strata is the intact layering within these mounds. A few, small mounds (type 2 – mounds resting on flat seafloor reflection) do not show “pop-up” structures or low-angled concave reflections beneath, and appear above a bright seafloor reflector (Fig. 3J, M4 and M2) and can be interpreted as depositional features or so called ejecta deposits. However, the brightening at the mounds´base can alternatively be caused by very shallow underlying gas/hydrate accumulations.

## Conclusions

We conclude that large craters in the northern Barents Sea are seafloor collapse structures oriented by down-thrown fault blocks and associated with gas hydrate dissociation. The distribution and shape of the collapse structures are defined by the fault and fracture pattern in the area. We suggest that bedrock collapse and methane emission phases were driven by underlying volume loss associated with gas hydrate dissociation, fluidization and gas escape during the last deglaciation. We further infer that gas hydrate growth and dissociation must have played an important role in mound and crater formation causing subsidence and collapse, in line with Andreassen *et al*., 2017. As follows, we suggest that a subglacial gas hydrate system leading to crater formation and extensive methane release upon ice sheet retreat some 15–12 ka BP was controlled by a pre-existing and reactivated tectonic fault and fracture systems, and so is the present gas seepage. Craters and mounds of similar sizes are found elsewhere in Arctic^3,5,10,13,14^ and Antarctic^11^ regions on current and buried seafloors, suggesting that large-scale methane emission phases also took place in other geographic regions and over longer time intervals. We infer that similar gas hydrate dynamics, formation of mounds, craters and methane ejections to the ocean and the atmosphere could take place in polar regions today where contemporary ice sheets are retreating over faulted bedrocks containing deeper carbon sources.

## Methods

The high-resolution P-Cable 3D seismic cube acquired in July 2015 onboard R/V Helmer Hanssen covers an area of 30 km^2^. The P-Cable 3D seismic system^41,42^ consists of an array of 14, 25 m long streamers with 8 receiver groups. We acquired the seismic lines with a 50% overlap at a sailing speed of ~4 knots. A shot was fired at ~160 bar every 6 second using a GI-airgun of 730/1700 cm^3^ operated in a Generator-Injector (GI) mode. This setup provides a lateral resolution of ~6 meters, and a frequency bandwidth between 10 and 400 Hz. The seismic processing was conducted in the RadexPro 2014 software package. The 3D processing flow consisted of following steps; (1) import of raw shot data in Seg-D format, (2) first-order denoising using a bandpass filter of 10-20-350-500 Hz to eliminate noise not related to the source signal, (3) inspected of additional noise on single channels, (4) navigational data applied to each trace using a calculated symmetric shaped cross-cable and adjusted to the first break, (5) merge of lines, (6) amplitude correction for geometric spreading, (7) time-correction for tidal variations, (8) assignment of common-depth-point with a 6.25 m bin-space, (9) deghosting using the SharpSeis method due to a prominent ghost appearing just beneath the seafloor, (10) NMO correction using a velocity of 1470 m/s and mean stacking, and lastly (11) interpolation and (12) post-stack kirschof migration and additional amplitude gain (Fig. S-2). Strong seafloor reflectivity combined with a high seismic frequency content produce a shallow seismic penetration limited to ~100–150 ms TWT below the seafloor (~100–150 m). The dominant seismic frequency of the seafloor is approximately 100 Hz and along the horizon at ~550 ms it is 80 Hz, implying an average vertical feature detectability, without filtering of ~5–6 m (Fig. S-2). The horizontal resolution is regarded as comparable to the bin size of 6.25 m. Depth converted surfaces are generated using a gradual increase of seismic p-wave velocity from seafloor at 1800 m/s to CA4 at 2200 m/s.

High-resolution 3D seismic is a unique tool for detailed horizon, fault segment and damage zone characterization that might play a key role controlling leakage pathways (see Jones and Knipe^43^). The 3D seismic interpretation and analysis is carried out using the seismic interpretation program Petrel 2014/15. Here we characterize the 3D architecture of craters and mounds, assess underlying structural and stratigraphic settings by visualizing and interpreting some of the most prominent seismic horizons, the faulted/fractured network and indications of subsurface fluids within the 3D seismic data. We applied ant-tracking on a variance volume^44^, and tested different parameters for the best result, however, discontinuities and noisy data made it impossible to extract faults automatically from the ant tracking volume. Therefore, faults are interpreted manually based on variance, ant tracking^45^ and Root-Mean-Square (RMS) amplitude volumes. We further perform fault seal analysis using various attributes to derive fault orientations, dips, and distance to faults, located footwalls and hanging walls, dimension of potential damage zones (areas of low seismic continuity), fault clustering characteristics and fault displacements.

Small displacement faults (<30 m) are frequently observed along the conventional 2D lines crossing the study area, as well as within the 3D data (Figs. 3, S-1, S-2). These small-displacement faults penetrate the clinoforms and often extend to the seafloor (Fig. S-1C). The high density of the faults makes it impossible to trace them between the available 2D lines, thus the areal high resolution 3D seismic is a unique dataset to extract proper fault information from.

Multibeam Echosounder data was collected using an EM300 system at a 30-kHz sound wave frequency and an angular coverage of 135°, which provided an average horizontal resolution of 5 m. Gas flare data are acquired from single-beam echosounder data using a Simrad EK 60 system with a 38-kHz signal. The system detects gas bubbles in the water column due to the high acoustic impedance contrast between gas and water.

## Supplementary information

Supplementary information.

## Data Availability

The data that support the findings of this study are available from the corresponding author upon reasonable request.
