# Author Correction: Geological controls of giant crater development on the Arctic seafloor

**DOI:** 10.1038/s41598-021-98947-0

**Published:** 2021-09-21

**Authors:** Malin Waage, Pavel Serov, Karin Andreassen, Kate A. Waghorn, Stefan Bünz

**Affiliations:** CAGE – Centre for Arctic Gas Hydrate, Environment, and Climate, Department of Geosciences, UiT the Arctic University of Norway, 9037 Tromsø, Norway

Correction to: *Scientific Reports* 10.1038/s41598-020-65018-9, published online 21 May 2020

The original version of this Article contained errors in Figure 2 where the longitude coordinates and the figure call-outs were incorrect in panel (A). The original Figure 2 and accompanying legend appear below.

**Figure 2:**
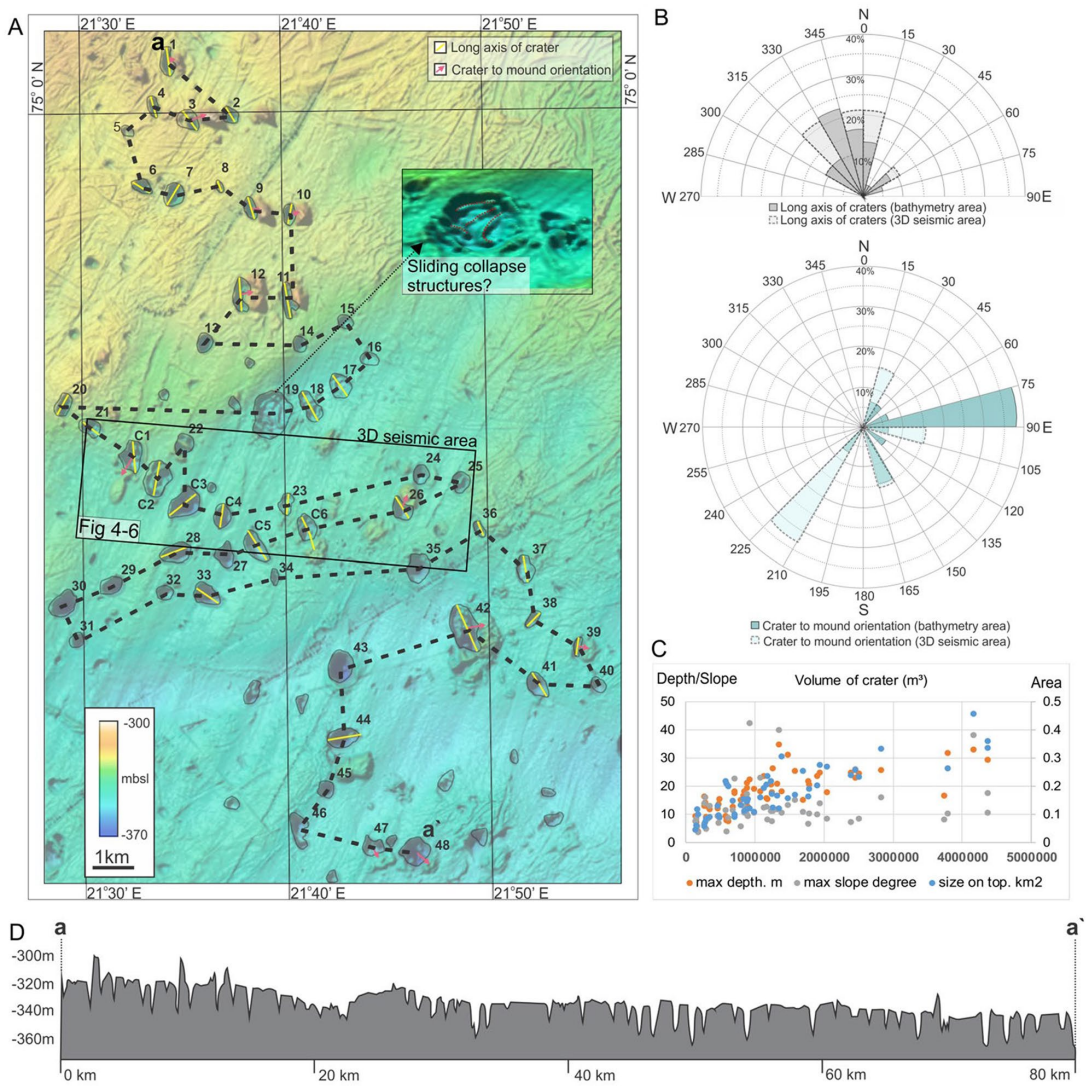
The study area with 54 large craters and 15 mounds. (**A**) bathymetric map. The yellow lines represent the long axis of craters and pink arrow the crater to mound orientation. The craters that show an asymmetry of > 1.6 are regarded as elongated, and included in the orientation measures. These orientations are presented in a rose diagram in inset (**B**). The data show a NNW trend of crater orientations, and that mounds tend to be located on the eastern side of the crater-mound couples. (**C**)-panel showing statistical data where crater volume (m^3^) is plotted against surface area (m^2^), maximum depth (m) and maximum slope (degrees). (**D**) profile through all craters.

The original Article has been corrected.

