# Supplementary material for: Geological controls of giant crater development on the Arctic seafloor
Source: Sci Rep. 2020 May 21;10:8450. doi: 10.1038/s41598-020-65018-9 (PMC7242475; doi:10.1038/s41598-020-65018-9)
Supplement: Supplementary file 1 — Supplementary information. [file 41598_2020_65018_MOESM1_ESM.docx]

# Geological controls of giant crater development on the Arctic seafloor

Malin Waage^1*^, Pavel Serov^1^, Karin Andreassen^1^, Kate A. Waghorn^1^, Stefan Bünz^1^

^1^CAGE – Centre for Arctic Gas Hydrate, Environment, and Climate, Department of Geosciences, UiT the Arctic University of Norway, 9037 Tromsø, Norway.

* Corresponding Author: Malin Waage [malin.waage@uit.no](mailto:malin.waage@uit.no)

### Supplementary Fig. S-1 Regional geology

### Supplementary Fig. S-2 Seismic data examples and a shallow borehole from the site.

**
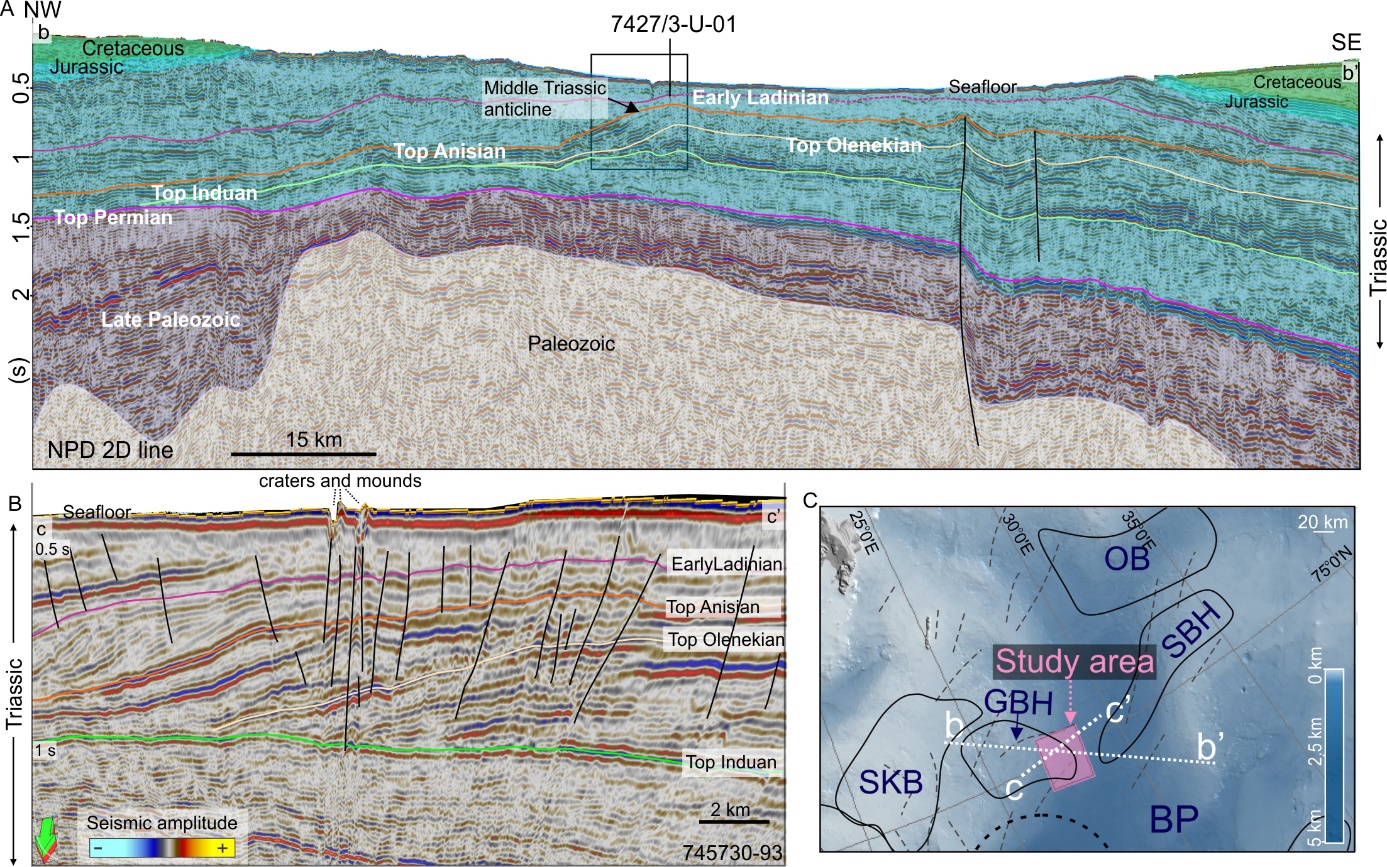
**

**Figure S-1.** A and B - Conventional 2D seismic lines across the study showing the shallow Triassic succession of clinoforms outcropping in the study area, a box outlining Fig 2B and the location of the shallow borehole. In B, we indicate the presence of faults penetrating the Early Ladinian-Top Induan succession. C shows the location of seismic line A and B.


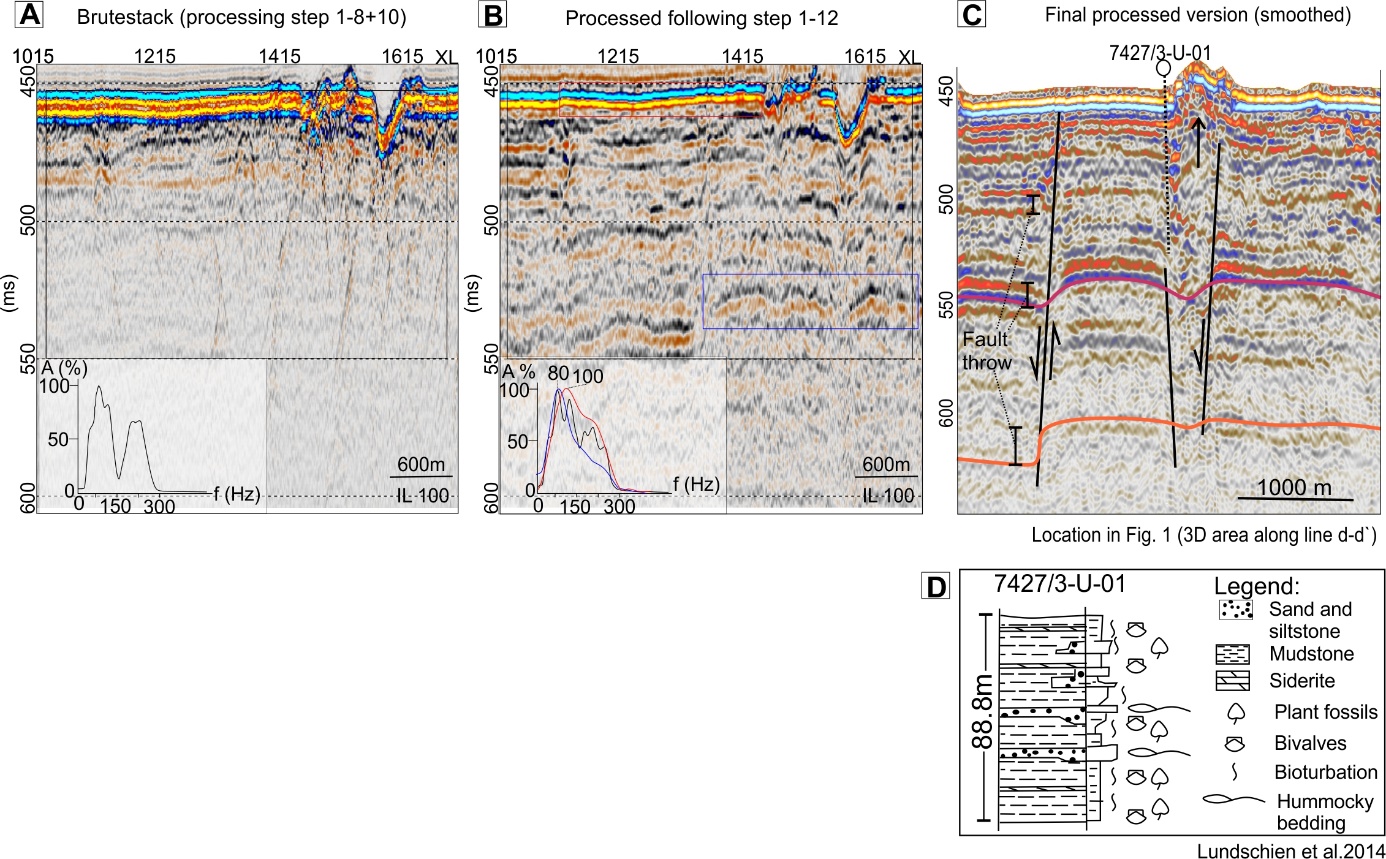


**Figure S-2.** Seismic and frequency spectrum example of the high-resolution 3D seismic data. A. brute-stack inline example processed following step 1-8 and 10 and B. finally processed inline example following processing step 1-12. C. shows a smoothed seismic section. The section illustrate decreasing fault displacement with decreasing depths.
